# New-Onset Bipolar Disorder in Late Life: a case report and review of literature

**DOI:** 10.1192/j.eurpsy.2023.1446

**Published:** 2023-07-19

**Authors:** A. Sanz Giancola, E. Arroyo Sánchez, P. Setién Preciados, M. Martín Velasco, I. Romero Gerechter, C. Díaz Mayoral

**Affiliations:** Psychiatry, Hospital Universitario Príncipe de Asturias, Alcalá de Henares, Spain

## Abstract

**Introduction:**

The elderly represents the fastest growing group of the population. It is fair to assume that the portion of old age patients suffering from bipolar disorder will grow in a similar manner. Elderly patients represent approximately 25% of the bipolar population. Summarizing, 5–10% of patients were 50 years of age when they experienced their first manic episode, constituting the subgroup of late onset bipolar disorder (LOBD).

**Objectives:**

The purpose of this case report and literature review is to emphasise the importance of LOBD in old population and to highlight its still sparse-knowledge.

**Methods:**

Descriptive case study and review of literature (Arnold,I. et al. Old Age Bipolar Disorder—Epidemiology, Aetiology and Treatment. Medicina **2021**,57,587; Baldessarini et al. Onset-age of bipolar disorders at six international sites. J Affect Disord 2010;121(1-2):143-6).

**Results:**

A 60-year-old woman is brought to the emergency department for evaluation by her family. Over the past 7 days, the patient has become increasingly irritable and argumentative, is sleeping less, is talking faster than usual and has begun to express paranoid concerns about her students “stealing my exam”. The patient is a university professor.

In the assessment interview she is hyperverbal, expansive, and grandiose. The family has also just recently discovered that she has spent a large sum of money on the Internet.

She has no history of psychiatric contact or substance use disorders; however, she has a family history of severe depression.

In the absence of any plausible non-psychiatric condition that could mimic or induce mania, the working diagnosis is bipolar I disorder, most recent episode (MRE) manic with psychotic features.

**Image:**

**Figure:**
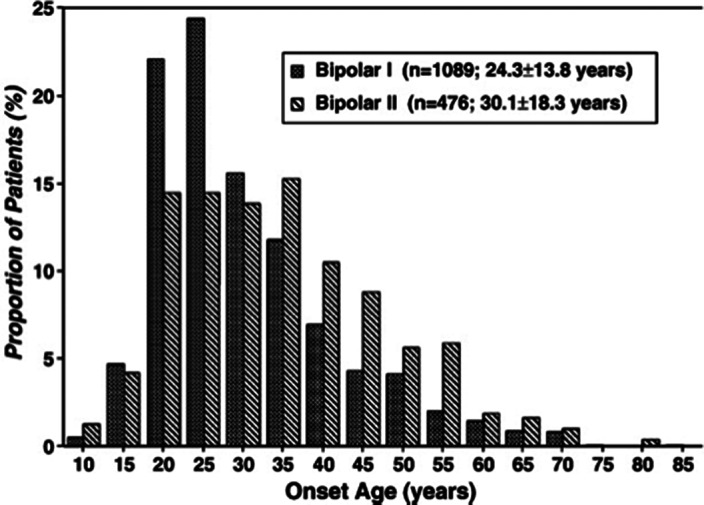

**Image 2:**

**Figure:**
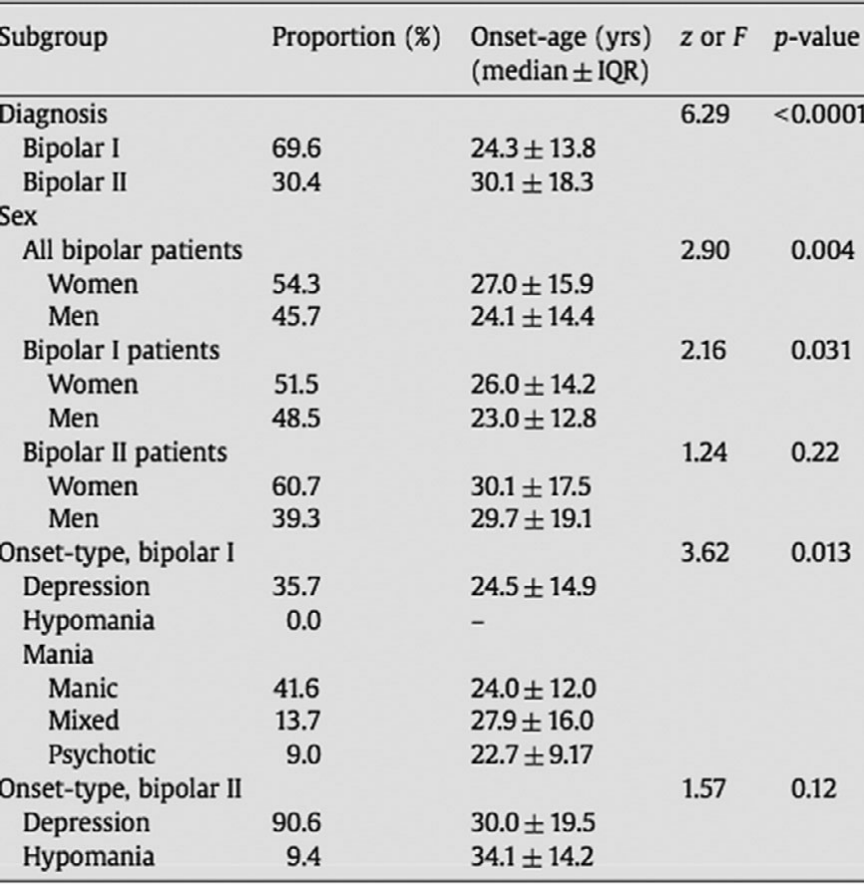

**Conclusions:**

The share of older age bipolar disorder will grow constantly in the next decades and further research on this neglected patient group is urgently required.

**Disclosure of Interest:**

None Declared

